# Glycopeptide Antibiotic Teicoplanin Inhibits Cell Entry of SARS-CoV-2 by Suppressing the Proteolytic Activity of Cathepsin L

**DOI:** 10.3389/fmicb.2022.884034

**Published:** 2022-04-28

**Authors:** Fei Yu, Ting Pan, Feng Huang, Ruosu Ying, Jun Liu, Huimin Fan, Junsong Zhang, Weiwei Liu, Yingtong Lin, Yaochang Yuan, Tao Yang, Rong Li, Xu Zhang, Xi Lv, Qianyu Chen, Anqi Liang, Fan Zou, Bingfeng Liu, Fengyu Hu, Xiaoping Tang, Linghua Li, Kai Deng, Xin He, Hui Zhang, Yiwen Zhang, Xiancai Ma

**Affiliations:** ^1^Guangdong Provincial People’s Hospital, Guangdong Academy of Medical Science, Guangzhou, China; ^2^Key Laboratory of Tropical Disease Control of Ministry Education, Guangdong Engineering Research Center for Antimicrobial Agent and Immunotechnology, Zhongshan School of Medicine, Institute of Human Virology, Sun Yat-sen University, Guangzhou, China; ^3^Center for Infection and Immunity Study, School of Medicine, Shenzhen Campus of Sun Yat-sen University, Shenzhen, China; ^4^Bioland Laboratory (Guangzhou Regenerative Medicine and Health Guangdong Laboratory), Guangzhou, China; ^5^Guangzhou Eighth People’s Hospital, Guangzhou Medical University, Guangzhou, China; ^6^Guangzhou Women and Children Medical Center, Guangzhou Institute of Pediatrics, Guangzhou, China; ^7^National Guangzhou Laboratory, Bio-Island, Guangzhou, China

**Keywords:** teicoplanin, SARS-CoV-2, spike, cathepsin L, viral entry

## Abstract

Since the outbreak of the coronavirus disease 2019 (COVID-19) caused by severe acute respiratory syndrome coronavirus 2 (SARS-CoV-2), public health worldwide has been greatly threatened. The development of an effective treatment for this infection is crucial and urgent but is hampered by the incomplete understanding of the viral infection mechanisms and the lack of specific antiviral agents. We previously reported that teicoplanin, a glycopeptide antibiotic that has been commonly used in the clinic to treat bacterial infection, significantly restrained the cell entry of Ebola virus, SARS-CoV, and MERS-CoV by specifically inhibiting the activity of cathepsin L (CTSL). Here, we found that the cleavage sites of CTSL on the spike proteins of SARS-CoV-2 were highly conserved among all the variants. The treatment with teicoplanin suppressed the proteolytic activity of CTSL on spike and prevented the cellular infection of different pseudotyped SARS-CoV-2 viruses. Teicoplanin potently prevented the entry of SARS-CoV-2 into the cellular cytoplasm with an IC_50_ of 2.038 μM for the Wuhan-Hu-1 reference strain and an IC_50_ of 2.116 μM for the SARS-CoV-2 (D614G) variant. The pre-treatment of teicoplanin also prevented SARS-CoV-2 infection in hACE2 mice. In summary, our data reveal that CTSL is required for both SARS-CoV-2 and SARS-CoV infection and demonstrate the therapeutic potential of teicoplanin for universal anti-CoVs intervention.

## Introduction

Coronaviruses (CoVs) are enveloped positive sense single-stranded RNA viruses (Cui et al., 2019; Chen et al., 2020). Many members of the coronavirus family are life-threatening human pathogens and can cause severe respiratory diseases, such as severe acute respiratory syndrome-associated coronavirus (SARS-CoV) that emerged in 2003 and the middle east respiratory syndrome coronavirus (MERS-CoV) that emerged in 2012 (Peiris et al., 2003a,b; Lau et al., 2005; Zaki et al., 2012; Chan et al., 2013; Lu et al., 2015; de Wit et al., 2016). Since December 2019, a novel coronavirus has emerged and spread globally, resulting in millions of pneumonia cases around the world (Horton, 2020; Huang et al., 2020; Lu et al., 2020; Rothe et al., 2020; Zhu et al., 2020). This novel coronavirus, named SARS-CoV-2, belongs to the beta-coronavirus according to the sequence released (Wu et al., 2020; Zhu et al., 2020). Evolutionary analyses have shown that SARS-CoV-2 shares 79% homology with SARS-CoV and 50% homology with MERS-CoV (Chan et al., 2020; Wu et al., 2020; Zhou et al., 2020). During the last 2 years, many independent dominant SARS-CoV-2 variants have emerged locally and circulated globally, which included B.1.1.7 (Alpha), B.1.351 (Beta), P.1 (Gamma), B.1.429 (Epsilon), B.1.525 (Eta), B.1.526 (Iota), B.1.617.1 (Kappa), B.1.617.2 (Delta), B.1.621 (Mu), C.37 (Lambda), and B.1.1.529 (Omicron) (Candido et al., 2020; Cherian et al., 2021; He et al., 2021; Kimura et al., 2021; Laiton-Donato et al., 2021; McCallum et al., 2021; Meng et al., 2021; Ozer et al., 2021; Tegally et al., 2021; Zhou et al., 2021). Given the high frequency of mutation, the high infectious rate, and the lack of effective treatment for SARS-CoV-2, it is urgent to develop an efficient antiviral drug for SARS-CoV-2 and its mutants.

The spike (S) glycoproteins, which cover on the surface of virions, mediate the viral entry into host cells and determine the host range of coronaviruses (Gallagher and Buchmeier, 2001; Ou et al., 2020; Shang et al., 2020). The infection of both SARS-CoV and SARS-CoV-2 is initiated by the attachment of the S protein to the host receptor angiotensin-converting enzyme 2 (ACE2) (Li et al., 2003; Hoffmann et al., 2020; Shang et al., 2020), followed by the S protein priming by cellular proteases such as TMPRSS2 (Glowacka et al., 2011; Shulla et al., 2011; Reinke et al., 2017; Hoffmann et al., 2020). In cells lacking TMPRSS2, SARS-CoV-2 can enter cells by endocytosis, during which, viruses are transported into host cells through the early endosomes and late endosomes, and subsequently endo/lysosomes. For SARS-CoV, the S proteins are further cleaved by other proteases such as cysteine proteinase cathepsin L (CTSL) within endocytic vesicles to complete the activation of S proteins (Matsuyama et al., 2005; Simmons et al., 2005; Huang et al., 2006; Belouzard et al., 2009). The activated S proteins then mediate the fusion of viral membranes and cellular membranes, resulting in the release of SARS-CoV genome into the cytoplasm and subsequent viral expression and replication.

We previously found that teicoplanin, a commonly used clinical glycopeptide antibiotic, potently suppressed the cellular entry of Ebola virus, SARS-CoV, and MERS-CoV (Zhou et al., 2016). Upon binding to the corresponding cellular receptors, the glycoproteins of Ebola virus and the Spike glycoproteins of both SARS-CoV and MERS-CoV could initiate the viral entry through endocytosis or micropinocytosis (Chandran et al., 2005; Simmons et al., 2005; Shirato et al., 2013). Within endosomes, the endosomal proteolysis of glycoproteins by CTSL was required for the full activation of these glycoproteins and the subsequent release of the viral genome. Further mechanism investigation revealed that teicoplanin blocked the virus entry by specifically inhibiting the proteolytic activity of CTSL on viral glycoproteins (Simmons et al., 2005; Wang et al., 2016; Zhou et al., 2016). Without CTSL-mediated glycoprotein activation, these viruses would be gradually degraded within the endosomes. These studies indicated the potential of teicoplanin as an effective drug for CTSL-dependent viral infection. In this study, we investigated the role of CTSL in SARS-CoV-2 entry and tested the inhibitory effect of teicoplanin and homologs on the viral entry process. We found that the cleavage sites of CTSL were highly conserved among the S sequences of various epidemic SARS-CoV-2 mutants and SARS-CoV. The loss of CTSL significantly crippled SARS-CoV-2 infection, while the overexpression of CTSL significantly increased the infectivity of SARS-CoV-2. Meanwhile, teicoplanin and dalbavancin, but not vancomycin, exhibited remarkable inhibitory activity toward the entry of SARS-CoV-2. Teicoplanin was able to inhibit the entry of all the major epidemic SARS-CoV-2 mutants. Further mechanism study indicated that teicoplanin inhibited SARS-CoV-2 entry by inhibiting the proteolytic activity of CTSL on S proteins. More importantly, teicoplanin inhibited the entry of SARS-CoV-2 viruses with an IC_50_ lower than 5 μM (2.038 μM for the original strain, 2.116 μM for the D614G variant). The pre-treatment of teicoplanin also prevented the infection of SARS-CoV-2 in mice models. Combined with our previous finding that teicoplanin inhibited the entry of SARS-CoV and MERS-CoV, our study reported here indicated that the CTSL inhibitor teicoplanin could be a universal anti-CoVs drug.

## Materials and Methods

### Cell Lines and Viruses

HEK293T (ATCC, CRL-3216), A549 (ATCC, CCL-185), Huh7 (JCRB, JCRB0403), Calu-3 (ATCC, HTB-55), and Vero E6 (ATCC, CRL-1586) cells were maintained in DMEM (Thermo Fisher Scientific, United States) supplemented with 10% FBS (Thermo Fisher Scientific, United States), 100 units/ml penicillin, and 100 μg/ml streptomycin (Thermo Fisher Scientific, United States) at 37°C and 5% CO_2_. These cells expressed considerable amounts of human angiotensin-converting enzyme 2 (hACE2). The HEK293T-hACE2^high^ cell line was generated by infecting wildtype HEK293T cells with lentiviruses which expressed hACE2. The hACE2-positive cells were sorted by fluorescence activated cell sorting (FACS) and confirmed by western blot with antibodies against hACE2. The HEK293T-hACE2^high^ cells were maintained as wildtype HEK293T cells. The HEK293T-hACE2^high^/TMPRSS2^high^ cells and the Calu-3-CTSL^high^ cells were constructed similarly. All cells have been tested for mycoplasma by PCR-based assay and confirmed to be mycoplasma-free (Mycoplasma-F: 5′-GGGAGCAAACAGGATTAGTATCCCT-3′; Mycoplasma-R:5′-TGCACCATCTGTCACTCTGTTACCCTC-3′).

The plasmid expressing the S of SARS-CoV-2 (D614; Wuhan-Hu-1, GISAID: EPI_ISL_402125) was purchased from Generay Biotech company (Shanghai, China) and inserted into the pcDNA3.1 vector. SARS-CoV-2 S/HIV-1 pseudotyped viruses were packaged by co-transfecting a lentiviral construct pHIV-Luciferase (Addgene plasmid # 21375), a packaging construct psPAX2 (Addgene plasmid # 12260) and plasmid expressing S proteins into HEK293T cells. The luciferase gene within the pHIV-Luciferase vector is under the control of the EF-1α promoter and can be expressed after pseudotyped virus infection. Thus, the pseudotyped virus system can be used to indicate the infection of SARS-CoV-2 S/HIV-1 pseudotyped viruses. The culture medium was replaced with fresh DMEM at 6 h post transfection. The pseudotyped viruses-containing supernatant was collected 48 h post transfection and filtered through 0.45 μm filters. The amounts of pseudotyped viruses were quantified by RT-qPCR assays with primers against the long-term repeat (LTR) sequences of viral RNAs (HIVTotal-F: 5′-CTGGCTAACTAGGGAACCCACTGCT-LTR) sequences of viral RNAs (HIVTotal-F: 5′-CTGGCTAACTAGGGAACCCACTGCT-3′; HIVTotal-R: 5′-GCTTCAGCAAGCCGAGTCCTGCGTC-3′). These RT-qPCR experiments were performed as biological triplicate. Pseudotyped viruses including SARS-CoV S/HIV-1 and VSV-G/HIV-1 were packaged and quantified as pseudotyped SARS-CoV-2 S/HIV-1 viruses. Different pseudotyped SARS-CoV-2 S/HIV-1 mutants were also packaged and quantified as above, the S proteins of which included those of G614 virus (SYSU-IHV, EPI_ISL_444969), B.1.1.7 (Alpha, GISAID: EPI_ISL_581117), B.1.351 (Beta, EPI_ISL_678597), P.1 (Gamma, EPI_ISL_792683), B.1.429 (Epsilon, EPI_ISL_1675148), B.1.525 (Eta, EPI_ISL_1093465), B.1.526 (Iota, EPI_ISL_1080752), B.1.617.1 (Kappa, EPI_ISL_1372093), B.1.617.2 (Delta, EPI_ISL_1337507), B.1.621 (Mu, EPI_ISL_1220045) and C.37 (Lambda, EPI_ISL_1534645).

Patient-derived SARS-CoV-2 D614 virus (Wuhan-Hu-1) was obtained from Guangdong Provincial Center for Disease Control and Prevention. The SARS-CoV-2 G614 virus (SYSU-IHV) was isolated from the sputum sample of a female admitted in Guangzhou Eighth People’s Hospital who was infected at Guangzhou by an African traveler in April 2020. Vero E6 cells were utilized to propagate these viruses.

### Pseudotyped Virus Infection Assay

Pseudotyped viruses including SARS-CoV S/HIV-1, SARS-CoV-2 S/HIV-1, VSV-G/HIV-1, and 10 different SARS-CoV-2 mutants were packaged as above. For viral infection in siRNA-mediated gene knock-down experiment, targeted genes in HEK293T cells were firstly knocked down by a mixture of three different siRNAs targeting each gene, followed by the infection of pseudotyped SARS-CoV-2 S/HIV-1 viruses 24 h post transfection. Another 24 h later, siRNA- and virus-treated cells were lysed and measured for the amounts of luciferase proteins by luciferase reporter assays which could represent the percentages of virus-infected cells.

For virus infection in protein overexpression experiment, wildtype HEK293T cells were firstly transfected with CTSL-expressing or hACE2-expressing plasmids, followed by infection with pseudotyped SARS-CoV-2 S/HIV-1 viruses. The amounts of luciferase within each group were measured utilizing luminometer (Promega, United States) 48 h post infection and represented as relative luminescence units.

For virus infection in drug treatment experiment, HEK293T-hACE2^high^ cells were incubated with serially diluted drugs including teicoplanin (Selleck, S1399, United States), dalbavancin (Selleck, S4848, United States), vancomycin (Selleck, S2575, United States), and camostat (Selleck, S2874, United States), and different pseudotyped viruses. The amounts of luciferase within each group were measured 48 h post infection.

### Wildtype Virus Infection Assay

HEK293T-hACE2^high^ cells were seeded in 12-well plates. Twenty-four hours post seeding, cells were co-incubated with SARS-CoV-2 D614 (Wuhan-Hu-1) virus and twofold serially diluted teicoplanin. Another 48 h post incubation, the supernatant in each group was collected and proceeded to RNA extraction with RNeasy Mini Kit (QIAGEN, 74104, Germany) according to the manufacturer’s instruction. SARS-CoV-2 viral RNA copies were determined by one-stepSARS-CoV-2 RNA detection kit (PCR-Fluorescence Probing) (Da An Gene Co., DA0931, China) with the following primers and probes: N-F (5′-CAGTAGGGGAACTTCTCCTGCT-3′), N-R (5′-CTTTGCTGCTGCTTGACAGA-3′), and N-P (5′-FAM-CTGGCAATGGCGGTGATGCTGC-BHQ1-3′). These RT-PCR experiments were performed as biological triplicate. The half maximal inhibitory concentration (IC50) of teicoplanin against SARS-CoV-2 D614 virus was calculated by GraphPad software (San Diego, United States) according to these viral RNA copies within each group. The IC50 of teicoplanin against SARS-CoV-2 G614 virus (SYSU-IHV) was determined similarly. SARS-CoV-2 virus infection assays were conducted in the BSL-3 facility of Sun Yat-sen University.

### Drug or Virus Pre-treatment Assay

To determine whether teicoplanin targeted the virus directly or targeted the host cell indirectly, HEK293T-hACE2^high^ cells were treated with drug or virus in three different ways. In the first group, HEK293T-hACE2^high^ cells were pre-treated with 0, 12.5, 25, and 50 μM teicoplanin, respectively. Four hours post drug treatment, cells were infected with pseudotyped SARS-CoV-2 S/HIV-1 virus. Another 48 h later, the amounts of luciferase within each group were monitored and represented as relative luminescence units. In the second group, HEK293T-hACE2^high^ cells were co-treated with different concentrations of teicoplanin and pseudotyped SARS-CoV-2 S/HIV-1 virus simultaneously. The amounts of luciferase were measured 48 h post co-treatment. In the third group, cells were pre-infected with pseudotyped SARS-CoV-2 S/HIV-1 virus. Four hours post infection, cells were treated with different concentrations of teicoplanin. Another 48 h later, cells were lysed and measured for the amounts of luciferase.

### *In vitro* Cathepsin L Enzymatic Inhibition Assay

To evaluate the CTSL enzymatic activity upon teicoplanin treatment *in vitro*, the purified 250 ng CTSL proteins (Sino Biological Inc., 10486-H08H, China) were added into the CTSL assay buffer (400 mM NaAc, 4 mM EDTA, 8 mM DTT, and pH 5.5) and incubated in ice for 15 min for CTSL activation. To evaluate the inhibition of teicoplanin on CTSL activity, the purified 250 ng CTSL proteins were also co-incubated with 50 μM teicoplanin (Selleck, S1399, United States). After activation, about 2 μg *in vitro* purified SARS-CoV S (Sino Biological Inc., 40634-V08B, China) or SARS-CoV-2 S (Sino Biological Inc., 40589-V08B1, China) were added into each group. S protein only group (without CTSL and teicoplanin) was set as control group. The S-CTSL-teicoplanin mixtures were incubated at 37°C for 1.5 h. The enzymatic reaction was stopped by adding with SDS-PAGE loading buffer and followed by boiling at 100°C for 10 min. Digested proteins were proceeded to SDS-PAGE and analyzed by silver staining (Sigma-Aldrich, PROTSIL2-1KT, United States).

### Animal Infection

Eight-week-old specific-pathogen-free (SPF) transgenic hACE2 mice (C57BL/6) were purchased from GemPharmatech Co., Ltd. (Cat No.: T037657). All mice were housed in SPF facilities at the Laboratory Animal Center of Sun Yat-sen University. Animal experiments were conducted in strict compliance with the guidelines and regulations of the Laboratory Monitoring Committee of Guangdong Province of China. The Ethics Committees of Guangdong Provincial People’s Hospital and Sun Yat-sen University approved the animal experiments.

Six hours before the viral challenge, four hACE2 mice were intraperitoneally administrated with 100 mg/kg body weight teicoplanin (dissolved in saline). Four hACE2 mice in the control group were intraperitoneally administrated with equal volume of saline. Six hours later, all the mice were intranasally challenged with 1 × 10^5^ focus-forming units (FFU) of SARS-CoV-2 D614 virus. Another 5 days later, mice were euthanized to harvest lung tissues. Viral RNA copies in lung tissues were quantified by one-step SARS-CoV-2 RNA detection kit (PCR-Fluorescence Probing) (Da An Gene Co., DA0931, China). These RT-PCR experiments were performed as biological quadruplicate. SARS-CoV-2 challenge studies were approved by the Ethics Committee of Zhongshan School of Medicine of Sun Yat-sen University on Laboratory Animal Care (Assurance Number: SYSU-IACUC-2021-B0020).

### Histopathology and Immunohistochemistry

Lung tissues of SARS-CoV-2-infected mice were fixed in 4% paraformaldehyde for at least 2 days. These lung tissues were embedded in paraffin and proceeded to histopathology and immunohistochemistry analysis (Nanjing FreeThinking Biotechnology Co., Ltd., China). For histopathology analysis, sections (3–4 μm) of lung tissues were stained with H&E. For immunohistochemistry analysis, sections of lung tissues were deparaffinized and rehydrated with xylene and gradient alcohol. Antigens were retrieved in citric acid buffer (pH 6.0) and quenched with 3% H_2_O_2_. After blocking with BSA, sections were incubated with rabbit anti-SARS-CoV-2 Nucleoprotein (N) for 24 h at 4°C, followed by incubating with goat anti-rabbit IgG secondary antibody (HRP-conjugated) and staining with 3,3′-diaminobenzidine. Antibody-conjugated sections were stained with hematoxylin, followed by dehydrating with gradient ethanol. Samples were covered by neutral balsam and imaged with HS6 microscope (Sunny Optical Technology Co., Ltd., China).

### Sequence Data Collection and Alignment

The genome sequences of SARS-CoV and SARS-CoV-2 were collected from the GenBank database^1^ and the GISAID’s EpiCoV database.^2^ The sequences of SARS-CoV circulating in 2003 contain 6 strains (accession numbers: AY278488, AY545918, AY545917, AY394977, AY394978, and AY394979). The sequences of SARS-CoV-2 include 13 variants: D614 (Wuhan-Hu-1, GISAID: EPI_ISL_402125), G614 (SYSU-IHV, EPI_ISL_444969), B.1.1.7 (Alpha, GISAID: EPI_ISL_581117), B.1.351 (Beta, EPI_ISL_678597), P.1 (Gamma, EPI_ISL_792683), B.1.429 (Epsilon, EPI_ISL_1675148), B.1.525 (Eta, EPI_ISL_1093465), B.1.526 (Iota, EPI_ISL_1080752), B.1.617.1 (Kappa, EPI_ISL_1372093), B.1.617.2 (Delta, EPI_ISL_1337507), B.1.621 (Mu, EPI_ISL_1220045), C.37 (Lambda, EPI_ISL_1534645), and B.1.1.529 (Omicron, EPI_ISL_6752027). The S gene sequences were obtained from the genome of SARS-CoV and SARS-CoV-2 according to the annotation in the GenBank database. The sequence datasets were aligned using the ClustalW program implemented in MEGA X software. Consensus sequences were created using BioEdit software^3^ based on the multiple alignment of SARS-CoV and SARS-CoV-2. The amino acid sequence logos were generated by WebLogo.

### Statistical Analysis

All the measurements in this study have been performed for at least three biological replicates by at least two lab technicians or students. Detailed statistical information including statistical tests, sample numbers, mean values, standard errors of the mean (SEM) and *p*-values have been shown in the main text and figure legends. Statistical analysis was conducted with Graphpad Prism 8.0 or Microsoft Excel. Biological triplicate and quadruplicate data were presented as mean ± SEM. A value of *p* ≥ 0.05 was not statistically significant and represented as “ns.” A value of *p* < 0.05 was statistically significant and represented as asterisk (*). A value of *p* < 0.01 was more statistically significant and represented as double asterisks (^**^). A value of *p* < 0.001 was the most statistically significant and represented as triple asterisks (^***^). When comparing mean differences between groups which were split by one independent variable, one-way ANOVA with Tukey’s multiple comparison test or Dunnett’s multiple comparison test was conducted. When comparing mean differences between groups which were split by two independent variables, two-way ANOVA with Tukey’s multiple comparisons test or Dunnett’s multiple comparisons test was conducted. For data with a normal distribution, we used Student’s *t*-test.

## Results

### Severe Acute Respiratory Syndrome Coronavirus 2 Infection Depended on the Activity of Cathepsin L

**FIGURE 1:**
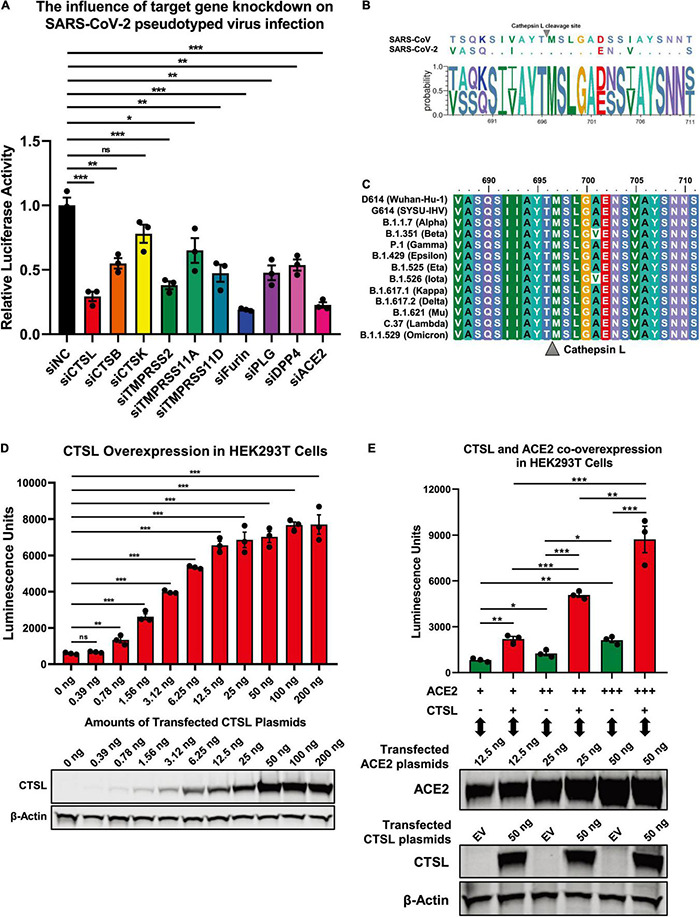
SARS-CoV-2 infection depended on the activity of CTSL. **(A)** CTSL, CTSB, CTSK, TMPRSS2, TMPRSS11A, TMPRSS11D, Furin, PLG, DPP4, and ACE2 in HEK293T cells were knocked down by siRNAs. These cells were infected with pseudotyped SARS-CoV-2 viruses 24 h post transfection. The intracellular luciferase activity was measured after another 24 h. The fold change of luciferase expression in each group was normalized to si-negative control (siNC) group (*n* = 3). **(B)** Sequence alignment based on the consensus spike (S) protein sequences of SARS-CoV and SARS-CoV-2. The overall height of the stack indicated the sequence conservation at that position, while the height of symbols within the stack indicated the relative frequency (Y-axis) of each amino acid at that position (X-axis). **(C)** The multiple alignments were created based on the region containing the cleavage site of cathepsin L (CTSL) (SIIAYTMSLGA) in the S protein of SARS-CoV-2. The S proteins of 13 different SARS-CoV-2 variants including D614 (Wuhan-Hu-1), G614 (SYSU-IHV), B.1.1.7 (Alpha), B.1.351 (Beta), P.1 (Gamma), B.1.429 (Epsilon), B.1.525 (Eta), B.1.526 (Iota), B.1.617.1 (Kappa), B.1.617.2 (Delta), B.1.621 (Mu), C.37 (Lambda), and B.1.1.529 (Omicron) were involved. The identity/similarity shading with the color refers to the chemistry of each amino acid at that position. **(D)** HEK293T cells in 96-well plate were transfected with twofold serially diluted CTSL-expressing plasmids, ranging from 0.39 to 200 ng. Cells were infected with pseudotyped SARS-CoV-2 S/HIV-1 viruses 24 h post transfection. Another 48 h post infection, cells were lysed and measured for the amounts of luciferase which were represented by luminescence units (*n* = 3). Western blot with antibodies against CTSL was conducted to confirm the expression of CTSL plasmids. β-Actin was immunoblotted as internal control. **(E)** HEK293T cells were transfected with different amounts of ACE2-expressing plasmids. The other groups of cells were co-transfected with CTSL-expressing plasmids. These cells were infected with pseudotyped SARS-CoV-2 S/HIV-1 viruses 24 h post transfection. The amounts of luciferase within each group were measured 48 h post infection and represented as luminescence units (*n* = 3). The expression of ACE2 and CTSL was confirmed by western blot. Data in **(A,D,E)** represented as mean ± SEM in triplicate. *P*-values in **(A,D)** were calculated by one-way ANOVA with Dunnett’s multiple comparison test which compared the mean of each group with the mean of the control group. *P*-values in **(E)** were calculated by one-way ANOVA with Tukey’s multiple comparison test which compared the mean of each group with the mean of every other group. ^ns^*p* ≥ 0.05, *p < 0.05, **p < 0.01, ***p < 0.001.

The proteolytic processing of the S protein is essential for SARS-CoV entry and fusion. Many host proteases, including TMPRSS2 and CTSL, are involved in the priming and activation of the SARS-CoV S protein, and some of which also have been identified and experimentally validated in SARS-CoV-2 infection (Bosch et al., 2008; Glowacka et al., 2011; Simmons et al., 2013; Hoffmann et al., 2020; Johnson et al., 2021; Wei et al., 2021; Zhao et al., 2021; Zhu et al., 2021). To systematically identify cellular proteases and receptors which mediated the entry and fusion of SARS-CoV-2 to target cells, we knocked down ten major proteases and receptors in HEK293T cells, which included CTSL, CTSB, CTSK, TMPRSS2, TMPRSS11A, TMPRSS11D, Furin, PLG, DPP4, and ACE2. Subsequently, these cells were infected with pseudotyped SARS-CoV-2 S/HIV-1 viruses which harbored an integrated *luciferase* gene. The expression of luciferase indicated the entry and expression of the pseudotyped virus. We found that the absence of CTSL, TMPRSS2, Furin, or ACE2 significantly decreased the pseudotyped SARS-CoV-2 virus infection (Figure 1A). The ACE2 protein has been identified as the major receptor of SARS-CoV-2 (Zhou et al., 2020). While the HEK293T cell line is a highly transfectable derivate of HEK293 cells, a cell line isolated from the kidney of a human embryo (HEK) that constitutively expresses the ACE2. Both TMPRSS2 and Furin also have been found to be essential for the efficient infection of SARS-CoV-2 (Hoffmann et al., 2020; Johnson et al., 2021). To determine whether CTSL is also involved in SARS-CoV-2 S protein activation, we first compared the cleavage sites of CTSL in the gene sequences encoding the SARS-CoV and SARS-CoV-2 S proteins. After alignment, we found that the cleavage sites of CTSL, the motif of which was AYT| MSL, were well-conserved between SARS-CoV and SARS-CoV-2 S proteins (Figure 1B). Moreover, the cleavage sites of CTSL on the S proteins were also highly conserved among all the major epidemic SARS-CoV-2 variants including D614 (Wuhan-Hu-1), G614 (SYSU-IHV), B.1.1.7 (Alpha), B.1.351 (Beta), P.1 (Gamma), B.1.429 (Epsilon), B.1.525 (Eta), B.1.526 (Iota), B.1.617.1 (Kappa), B.1.617.2 (Delta), B.1.621 (Mu), C.37 (Lambda), and B.1.1.529 (Omicron) (Candido et al., 2020; Cherian et al., 2021; He et al., 2021; Kimura et al., 2021; Laiton-Donato et al., 2021; McCallum et al., 2021; Meng et al., 2021; Ozer et al., 2021; Tegally et al., 2021; Zhou et al., 2021; Figure 1C). Previously, CTSL has been found to play pivotal roles in SARS-CoV infection by cleaving AYT| MSL motif within S proteins, which resulted in the activation of S proteins (Simmons et al., 2005; Bosch et al., 2008; Belouzard et al., 2012). We speculated that CTSL might also participate in SARS-CoV-2 entry and fusion utilizing the same proteolytic mechanism. Thus, we overexpressed CTSL proteins in HEK293T cells which were subsequently infected with pseudotyped SARS-CoV-2 S/HIV-1 viruses. We found that the infectivity of pseudotyped virus to HEK293T cells was linearly and positively correlated with the expression level of CTSL (Figure 1D). We also co-overexpressed ACE2 with CTSL in HEK293T cells. We found that the co-overexpression of CTSL significantly increased the infectivity of pseudotyped virus compared with ACE2-overexpression only (Figure 1E). These results indicated that the effective infection of SARS-CoV-2 to host cells depended on the proteolytic activity of CTSL.

### Teicoplanin Specifically Inhibited the Entry of Pseudotyped Severe Acute Respiratory Syndrome Coronavirus 2

**FIGURE 2:**
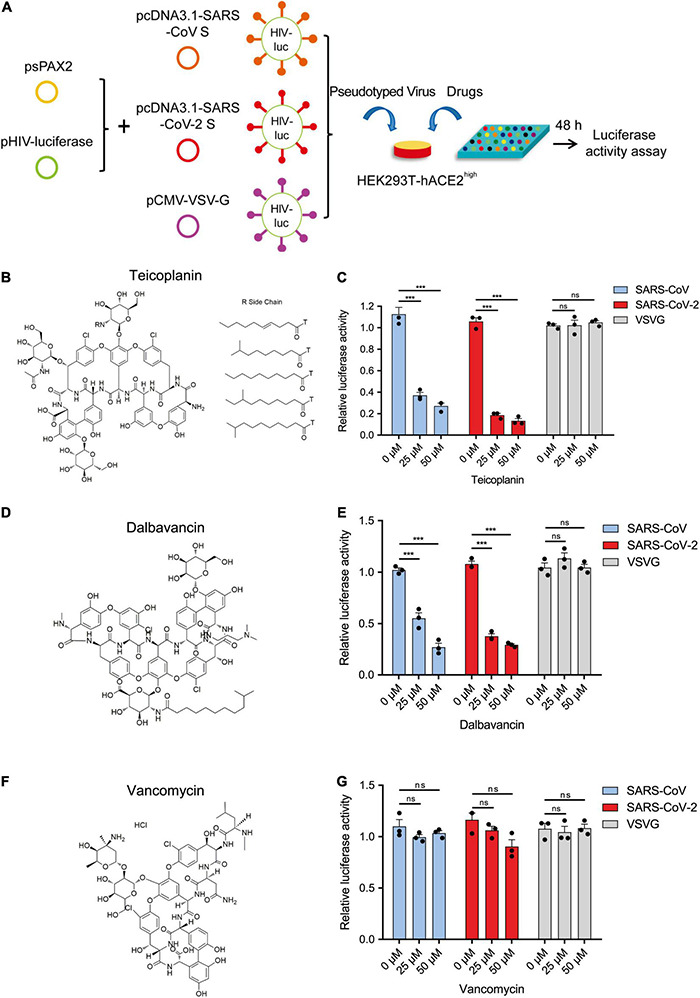
Teicoplanin specifically inhibited the entry of pseudotyped SARS-CoV-2. **(A)** Schematic of the pseudotyped virus entry upon drug treatment assay. To package different pseudotyped viruses, psPAX2 plasmids, and pHIV-Luciferase plasmids were co-transfected into HEK293T cells with pcDNA3.1-SARS-CoV S, pcDNA3.1-SARS-CoV-2 S, and pCMV-VSV-G plasmids, respectively. HEK293T-hACE2^high^ cells were incubated with drugs and different pseudotyped viruses. The amounts of luciferase within cells were measured 48 h post infection. **(B)** Chemical structure of teicoplanin. **(C)** HEK293T-hACE2^high^ cells were treated with 0, 25, and 50 μM teicoplanin, respectively, followed by infecting with different pseudotyped viruses including SARS-CoV S/HIV-1, SARS-CoV-2 S/HIV-1, and VSV-G/HIV-1. The intracellular luciferase activity was measured 48 h post infection (*n* = 3). **(D)** Chemical structure of dalbavancin. **(E)** HEK293T-hACE2^high^ cells were treated as in **(C)**, except that the drug was replaced with dalbavancin (*n* = 3). **(F)** Chemical structure of vancomycin. **(G)** HEK293T-hACE2^high^ cells were treated as in **(C)**, except that the drug was replaced with vancomycin (*n* = 3). Data in **(C,E,G)** represented as mean ± SEM in triplicate. *P*-values were calculated by two-way ANOVA with Dunnett’s multiple comparisons test which compared the mean of each group with the mean of the control group. ^ns^*p* ≥ 0.05, ***p < 0.001.

Previously, we have found that teicoplanin, a glycopeptide antibiotic which inhibited CTSL activity, suppressed the entry of SARS-CoV, MERS-CoV, and Ebola viruses (Zhou et al., 2016). We speculated that teicoplanin might also be able to block the entry of SARS-CoV-2. Thus, we conducted the pseudotyped virus entry upon drug treatment assay. We generated a highly sensitive HEK293T-hACE2^high^ cell line which constitutively expressed high level of hACE2 receptors. HEK293T-hACE2^high^ cells were co-incubated with teicoplanin and pseudotyped SARS-CoV-2 S/HIV-1 virus. The infectivity of pseudotyped virus, which was represented by the amounts of luciferase within HEK293T-hACE2^high^ cells, was measured 48 h post infection (Figure 2A). To exclude the possibility that teicoplanin inhibited the early events of the pseudotyped HIV-1 life cycle, pseudotyped VSV-G/HIV-1 viruses bearing vesicular stomatitis virus (VSV) glycoproteins were also packaged and treated as the negative control. Pseudotyped SARS-CoV S/HIV-1 viruses bearing SARS-CoV S were packaged and treated as the positive control. The results showed that teicoplanin effectively inhibited the entry of both SARS-CoV-2 and SARS-CoV pseudotyped viruses in a dose-dependent manner, whereas teicoplanin treatment did not affect the infection of pseudotyped VSV-G/HIV-1 viruses (Figures 2B,C).

Teicoplanin homologs including dalbavancin also have specific inhibitory effects on CTSL based on our previous study (Zhou et al., 2016). While vancomycin, another glycopeptide antibiotic which was clinically used for Gram-positive bacterial infections, did not show inhibitory activity on CTSL. Therefore, we further tested whether dalbavancin and vancomycin could inhibit the entry of SARS-CoV-2. Similar to teicoplanin, dalbavancin effectively inhibited both SARS-CoV-2 and SARS-CoV pseudotyped viruses entering into HEK293T-hACE2^high^ cells in a dose-dependent manner, but it did not affect pseudotyped VSV-G/HIV-1 virus infection (Figures 2D,E). In contrast, vancomycin did not show any inhibitory activity on the infection of pseudotyped SARS-CoV-2, SARS-CoV, or VSV-G viruses (Figures 2F,G). Taken together, these results indicated that CTSL inhibitors teicoplanin and its homolog dalbavancin could suppress the entry of SARS-CoV-2.

### Teicoplanin Inhibited the Entry of Authentic Severe Acute Respiratory Syndrome Coronavirus 2

**FIGURE 3:**
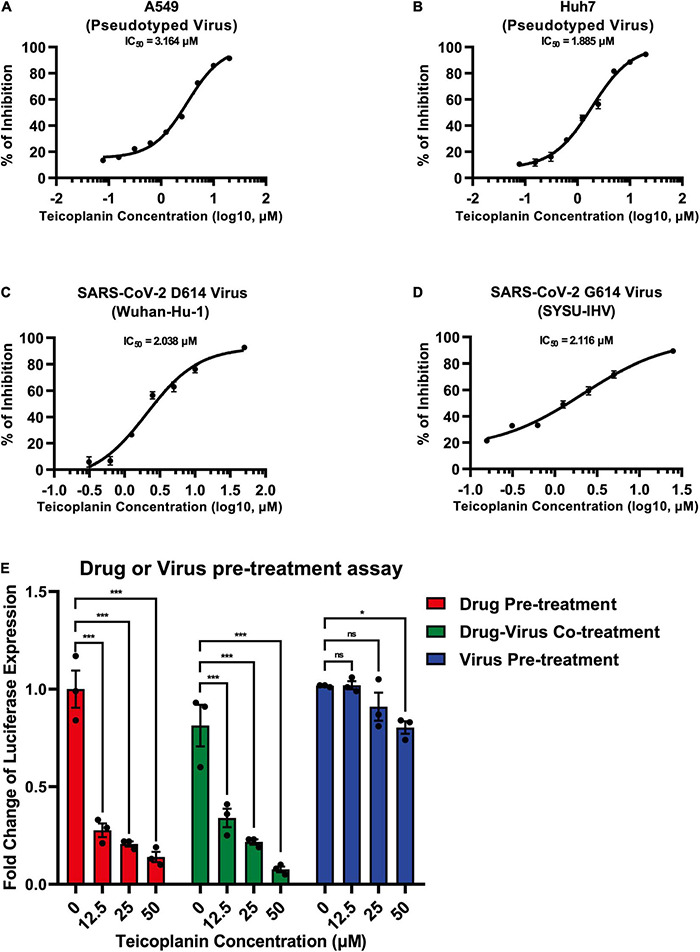
Teicoplanin inhibited the entry of authentic SARS-CoV-2. **(A)** A549 cells were co-incubated with pseudotyped SARS-CoV-2 S/HIV-1 viruses and twofold serially diluted teicoplanin. The expression of luciferase within cells were measured 48 h post incubation. The IC_50_ of teicoplanin against pseudotyped SARS-CoV-2 S/HIV-1 in A549 cells was calculated according to the relative expression of luciferase (*n* = 3). **(B)** The IC_50_ of teicoplanin against pseudotyped SARS-CoV-2 S/HIV-1 in Huh7 cells was calculated as in **(A)** (*n* = 3). **(C)** HEK293T-hACE2^high^ cells were co-incubated with SARS-CoV-2 D614 (Wuhan-Hu-1) virus and twofold serially diluted teicoplanin. At 48 h post incubation, the supernatant in each group was collected and proceeded to RNA extraction. Viral RNA copies in supernatant were quantified by one-step SARS-CoV-2 RNA detection kit. The IC_50_ of teicoplanin against SARS-CoV-2 D614 virus was calculated according to these viral RNA copies within each group (*n* = 3). **(D)** The IC_50_ of teicoplanin against SARS-CoV-2 G614 (SYSU-IHV) virus was determined as in **(C)** (*n* = 3). **(E)** In the first group, HEK293T-hACE2^high^ cells were pre-treated with 0, 12.5, 25, and 50 μM teicoplanin, respectively. Four hours later, cells were infected with pseudotyped SARS-CoV-2 S/HIV-1 virus. In the second group, cells were co-treated with different concentrations of teicoplanin and pseudotyped SARS-CoV-2 S/HIV-1 virus simultaneously. In the third group, cells were pre-infected with pseudotyped SARS-CoV-2 S/HIV-1 virus. Four hours post infection, cells were treated with different concentrations of teicoplanin. The amounts of luciferase within cells were quantified 48 h post infection. The fold changes of luciferase expression within each sample were calculated by normalizing to those in cells treated with 0 μM teicoplanin (*n* = 3). Data in **(A–D)** represented as mean ± SEM in triplicate. Inhibition curves in **(A–D)** were generated by log (inhibitor) vs. response non-linear fit. *P*-values in **(E)** were calculated by two-way ANOVA with Dunnett’s multiple comparisons test which compared the mean of each group with the mean of the control group. ^ns^*p* ≥ 0.05, *p < 0.05, ***p < 0.001.

To evaluate whether teicoplanin also inhibited SARS-CoV-2 infection in other susceptible cell lines, we infected A549 cells and Huh7 cells with pseudotyped SARS-CoV-2 S/HIV-1 viruses, accompanied by the treatment of serially diluted teicoplanin. The results showed that teicoplanin also effectively inhibited pseudotyped SARS-CoV-2 S/HIV-1 virus infection in both cell lines with a half maximal inhibitory concentration (IC_50_) of 3.164 μM for the A549 cell line and an IC_50_ of 1.885 μM for the Huh7 cell lines (Figures 3A,B). To further confirm that teicoplanin inhibited the entry of SARS-CoV-2, we investigated the antiviral activity of teicoplanin on authentic (live) SARS-CoV-2. We obtained two SARS-CoV-2 strains. One was SARS-CoV-2 D614 virus (Wuhan-Hu-1) which was provided by Guangdong Provincial Center for Disease Control and Prevention (GDCDC). The other was SARS-CoV-2 G614 virus (SYSU-IHV) which was isolated by us from the sputum sample of an infected patient (Ma et al., 2020). We found that teicoplanin effectively inhibited the entry of both strains with an IC_50_ of 2.038 μM for the Wuhan-Hu-1 reference strain and an IC_50_ of 2.116 μM for the SARS-CoV-2 (D614G) variant (Figures 3C,D). Given that the serum concentrations of teicoplanin in patients are at least 15 mg/L (8.78 μM) after the loading dose treatment for most Gram-positive bacterial infections, our data indicated that teicoplanin was able to potently suppress the entry of SARS-CoV-2 of both the original Wuhan-Hu-1 strain and the D614G mutation strain at a relatively low and safe dose (Zhou et al., 2016).

To elucidate whether the target of teicoplanin was the virus itself, or the host cell, or both, we conducted drug/virus pre-treatment assay. In the first group, HEK293T-hACE2^high^ cells were pre-treated with different concentrations of teicoplanin followed by infection with pseudotyped SARS-CoV-2 (drug pre-treatment group). In the second group, cells were co-incubated with both teicoplanin and pseudotyped virus (drug-virus co-treatment group). In the third group, cells were pre-infected with pseudotyped virus followed by treatment with teicoplanin (virus pre-treatment group). We found that the infectivity of pseudotyped SARS-CoV-2 in both drug pre-treatment group and drug-virus co-treatment group was negatively correlated with the concentration of teicoplanin, whereas the infectivity of pseudotyped virus in the virus pre-treatment group was almost unchanged upon the treatment of different concentrations of teicoplanin (Figure 3E). These results indicated that teicoplanin targeted host cells rather than viral particles.

### Teicoplanin Inhibited the Activity of Cathepsin L

Previous reports have shown that TMPRSS2 can activate S proteins for complete fusion through the cell surface entry route (Laporte et al., 2021). While CTSL activates S proteins to drive the fusion of viral membranes and endosomal membranes through the endosomal entry route. To identify whether TMPRSS2 and CTSL contributed to SARS-CoV-2 infection independently, we knocked down CTSL and TMPRSS2 in two different cell lines. One was HEK293T-hACE2^high^ cell line, which expressed high level of CTSL and low level of TMPRSS2 (Hoffmann et al., 2020). The other was Calu-3 cell line, which expressed high level of TMPRSS2 and low level of CTSL (Laporte et al., 2021). We found that the depletion of CTSL completely aborted the infection of pseudotyped SARS-CoV-2 S/HIV-1 viruses in CTSL^+^/TMPRSS2^–^ HEK293T-hACE2^high^ cells, whereas the depletion of TMPRSS2 did not abort the infection of pseudotyped SARS-CoV-2 S/HIV-1 viruses in the same cell line (Supplementary Figure 1A). The combination treatment of siCTSL and siTMPRSS2 also aborted the infection in HEK293T-hACE2^high^ cells, similar to the sole treatment of siCTSL. However, the depletion of CTSL in CTSL^–^/TMPRSS2^+^ Calu-3 cells only slightly decreased the infection of pseudotyped SARS-CoV-2 S/HIV-1 viruses (Supplementary Figure 1B). The depletion of TMPRSS2 as well as the co-treatment of siCTSL and siTMPRSS2 could significantly abort the infection of pseudotyped SARS-CoV-2 in Calu-3 cells (Supplementary Figure 1B). These results indicated that CTSL and TMPRSS2 contributed to the SARS-CoV-2 infection in different cell types.

**FIGURE 4:**
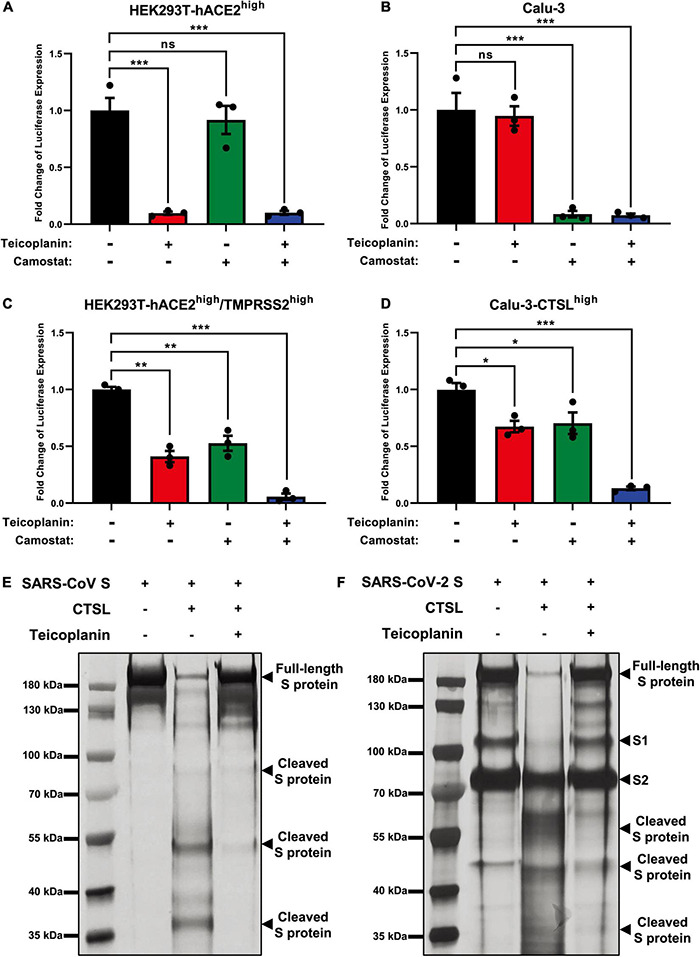
Teicoplanin inhibited the activity of CTSL. **(A)** HEK293T-hACE2^high^ cells were incubated with 25 μM teicoplanin or 50 μM camostat, followed by infecting with pseudotyped SARS-CoV-2 S/HIV-1 virus. The expression of luciferase was evaluated 48 h post infection (*n* = 3). **(B)** Calu-3 cells were incubated with 25 μM teicoplanin or 50 μM camostat, followed by infecting with pseudotyped SARS-CoV-2 S/HIV-1 virus. The fold change of luciferase expression was calculated as in **(A)** (*n* = 3). **(C)** HEK293T-hACE2^high^ cells were transfected with TMPRSS2-expressing plasmids to construct the HEK293T-ACE2^high^/TMPRSS2^high^ cell line. These cells were treated with 25 μM teicoplanin or 50 μM camostat, followed by infecting with pseudotyped SARS-CoV-2 S/HIV-1 virus. The fold change of luciferase expression was calculated as in **(A)** (*n* = 3). **(D)** Calu-3 cells were transfected with CTSL-expressing plasmids to construct the Calu-3-CTSL^high^ cell line. These cells were treated with 25 μM teicoplanin or 50 μM camostat, followed by infection with pseudotyped SARS-CoV-2 S/HIV-1 virus. The fold change of luciferase expression was calculated as in **(A)** (*n* = 3). **(E,F)** The *in vitro* purified 250 ng CTSL proteins were added into CTSL assay buffer for activation. In another group, CTSL were co-incubated with 50 μM teicoplanin. After activation, the *in vitro* purified SARS-CoV S or SARS-CoV-2 S proteins were added into each group. S protein only group was set as control group. The digested proteins were proceeded to SDS-PAGE and analyzed by silver staining. Data in **(A,D)** represented as mean ± SEM in triplicate. *P*-values were calculated by one-way ANOVA with Dunnett’s multiple comparison test which compared the mean of each group with the mean of the control group. ^ns^*p* ≥ 0.05, *p < 0.05, **p < 0.01, ***p < 0.001.

Our previous report has revealed that teicoplanin targets on CTSL directly within host cells (Zhou et al., 2016). While camostat inhibits the activity of TMPRSS2 (Kawase et al., 2012). To evaluate whether teicoplanin and camostat inhibited SARS-CoV-2 infection in different cell types, we firstly treated CTSL^+^/TMPRSS2^–^ HEK293T-hACE2^high^ cells with teicoplanin, camostat, or both. We found that the treatment of teicoplanin or the co-treatment of teicoplanin and camostat could significantly abort pseudotyped SARS-CoV-2 S/HIV-1 virus infection (Figure 4A). However, the sole treatment of camostat was unable to prevent the infection of pseudotyped SARS-CoV-2 in HEK293T-hACE2^high^ cells (Figure 4A). On the contrary, the sole treatment of camostat or the co-treatment of teicoplanin and camostat significantly inhibited pseudotyped SARS-CoV-2 S/HIV-1 virus infection in CTSL^–^/TMPRSS2^+^ Calu-3 cells (Figure 4B). While the sole treatment of teicoplanin was unable to inhibit the infection of pseudotyped SARS-CoV-2 in Calu-3 cells (Figure 4B). To further confirm that teicoplanin and camostat inhibited SARS-CoV-2 infection by targeting CTSL and TMPRSS2, respectively, we constructed an HEK293T-hACE2^high^/TMPRSS2^high^ cell line which expressed a high level of TMPRSS2. We found that neither teicoplanin nor camostat could totally abort pseudotyped SARS-CoV-2 S/HIV-1 virus infection in HEK293T-hACE2^high^/TMPRSS2^high^ cells (Figure 4C). While the combination of teicoplanin and camostat was able to significantly inhibit the infection of pseudotyped SARS-CoV-2 in the same cell line (Figure 4C). We also constructed a Calu-3-CTSL^high^ cell line which expressed a high level of CTSL. Similarly, only when the Calu-3-CTSL^high^ cells were co-treated with both teicoplanin and camostat could totally abort pseudotyped SARS-CoV-2 S/HIV-1 virus infection (Figure 4D). The sole use of teicoplanin or camostat could only partially inhibit the infection of pseudotyped SARS-CoV-2 (Figure 4D). Overall, teicoplanin and camostat inhibited SARS-CoV-2 infection by inhibiting the activity of CTSL and TMPRSS2, respectively.

To provide direct evidence that teicoplanin inhibiting SARS-CoV-2 entry *via* inhibiting the activity of CTSL, we conducted *in vitro* CTSL enzymatic inhibition assay. The *in vitro* purified CTSL proteins were firstly activated in CTSL assay buffer, followed by incubating with SARS-CoV S or SARS-CoV-2 S proteins (Supplementary Figures 2A–C). In another group, pre-activated CTSL proteins were co-incubated with different S proteins and teicoplanin. Then the CTSL- and teicoplanin-treated S proteins were proceeded to SDS-PAGE and silver staining. We found that CTSL proteins were able to effectively cleave both SARS-CoV S and SARS-CoV-2 S (Figures 4E,F). However, the co-treatment of teicoplanin with CTSL proteins inhibited the enzymatic activity of CTSL on both S proteins, resulting in the presence of more full-length S proteins (Figures 4E,F). Our above results further confirmed that teicoplanin inhibited the entry of SARS-CoV-2 by directly inhibiting the proteolytic activity of CTSL within host cells.

### Teicoplanin Inhibited the Entry of Various Severe Acute Respiratory Syndrome Coronavirus 2 Mutants

**FIGURE 5:**
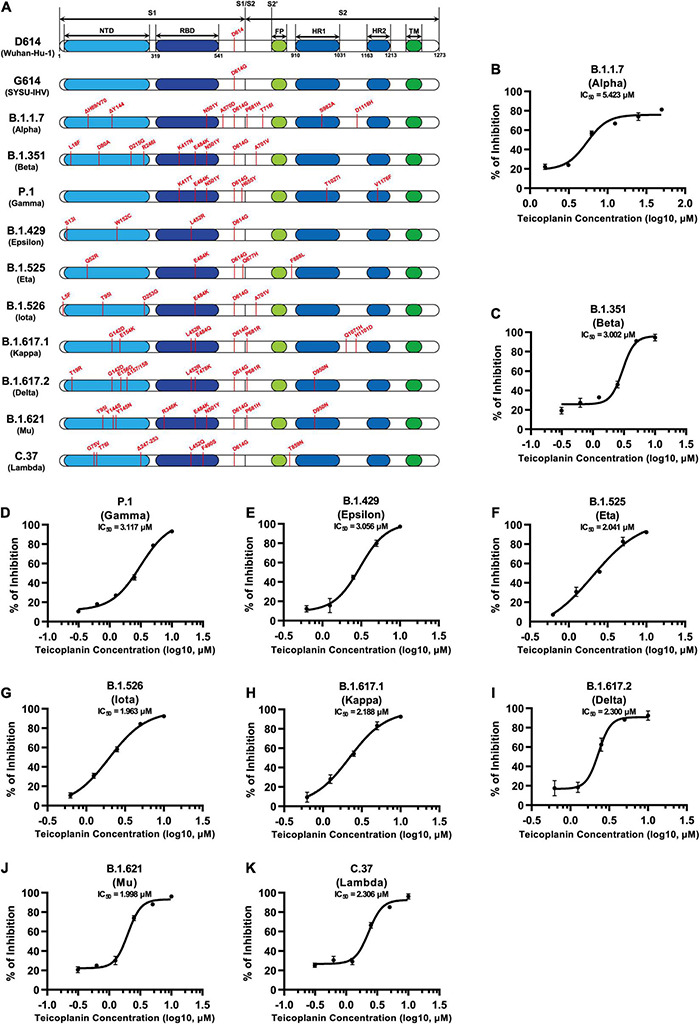
Teicoplanin inhibited the entry of various SARS-CoV-2 mutants. **(A)** Schematics of the S proteins of 12 different SARS-CoV-2 mutants which included D614 (Wuhan-Hu-1), G614 (SYSU-IHV), B.1.1.7 (Alpha), B.1.351 (Beta), P.1 (Gamma), B.1.429 (Epsilon), B.1.525 (Eta), B.1.526 (Iota), B.1.617.1 (Kappa), B.1.617.2 (Delta), B.1.621 (Mu), and C.37 (Lambda). The mutation sites were shown alongside each backbone and indicated in red. **(B–K)** HEK293T-hACE2^high^ cells were co-incubated with different pseudotyped SARS-CoV-2 S/HIV-1 viruses and serially diluted teicoplanin. The amounts of luciferase within each group were measured 48 h post infection and represented as luminescence units. The IC_50_ of teicoplanin against these pseudotyped SARS-CoV-2 mutants was calculated based on the amounts of luciferase within each group (*n* = 3). Data in **(B–K)** represented as mean ± SEM in triplicate. Inhibition curves were generated by log (inhibitor) vs. response non-linear fit.

Since December 2019, many SARS-CoV-2 mutants have emerged locally and spread worldwide, such as B.1.1.7 (Alpha), B.1.351 (Beta), P.1 (Gamma), B.1.429 (Epsilon), B.1.525 (Eta), B.1.526 (Iota), B.1.617.1 (Kappa), B.1.617.2 (Delta), B.1.621 (Mu), C.37 (Lambda), and B.1.1.529 (Omicron) (Candido et al., 2020; Cherian et al., 2021; He et al., 2021; Kimura et al., 2021; Laiton-Donato et al., 2021; McCallum et al., 2021; Meng et al., 2021; Ozer et al., 2021; Tegally et al., 2021; Zhou et al., 2021). We have found that the cleavage sites of CTSL on S proteins of these mutants were highly conserved (Figure 1C). Thus, we speculated that teicoplanin-mediated inhibition of CTSL activity might also cripple the cell entry of SARS-CoV-2 mutants. To evaluate whether teicoplanin still was able to inhibit the infection of these variants, we constructed different S-expressing plasmids which were derived from various SARS-CoV-2 mutants (Figure 5A). Like the package of pseudotyped SARS-CoV-2 (D614) S/HIV-1 viruses, we packaged ten different pseudotyped SARS-CoV-2 viruses based on the above S mutants. HEK293T-hACE2^high^ cells were co-incubated with different pseudotyped viruses and twofold serially diluted teicoplanin, followed by the measurement of the luciferase activity which could represent viral infectivity. The IC_50_ of teicoplanin against different pseudotyped viruses was calculated based on the percentages of viral inhibition. We found that all the IC_50_ of teicoplanin against the entry of these viruses were below 5 μM (3.002 μM for B.1.351/Beta, 3.117 μM for P.1/Gamma, 3.056 μM for B.1.429/Epsilon, 2.041 μM for B.1.525/Eta, 1.963 μM for B.1.526/Iota, 2.188 μM for B.1.617.1/Kappa, 2.300 μM for B.1.617.2/Delta, 1.998 μM for B.1.621/Mu, 2.306 μM for C.37/Lambda), except that the IC_50_ of teicoplanin against pseudotyped SARS-CoV-2 (B.1.1.7/Alpha) S/HIV-1 viruses was 5.423 μM (Figures 5B–K). Taken together, our above results indicated that the CTSL inhibitor teicoplanin was able to inhibit the entry of different SARS-CoV-2 variants.

### Teicoplanin Prevented Severe Acute Respiratory Syndrome Coronavirus 2 Infection in hACE2 Mice

**FIGURE 6:**
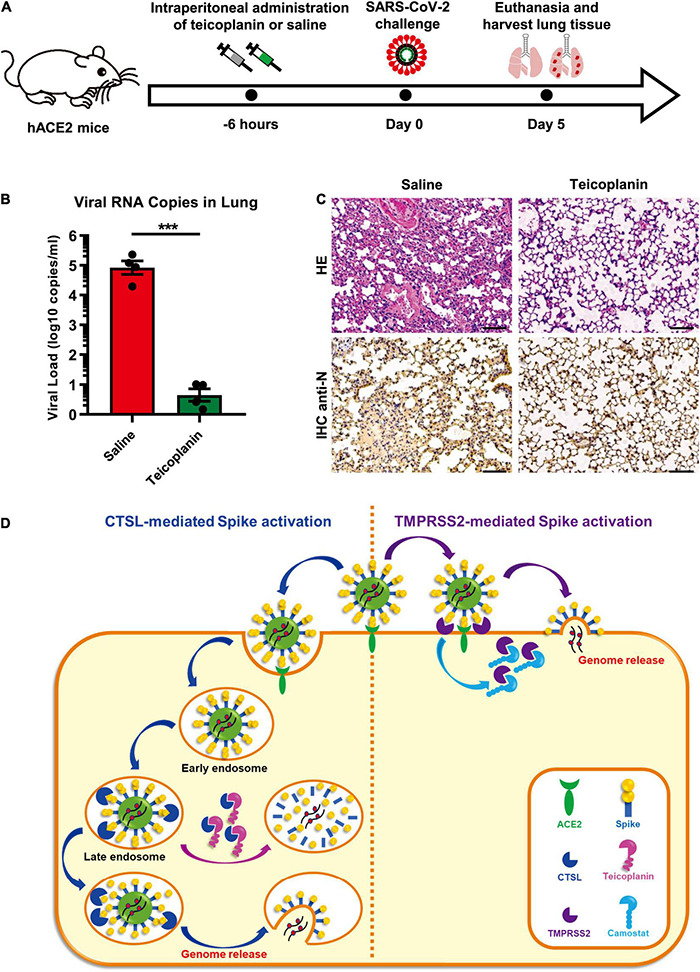
Teicoplanin prevented SARS-CoV-2 infection in hACE2 mice. **(A)** Schematic of mice experiment procedure. hACE2 mice were intraperitoneally administrated with 100 mg/kg body weight teicoplanin or saline (*n* = 4 in each group). Six hours later, each mouse was challenged with 1 × 10^5^ FFU of SARS-CoV-2 D614 viruses. Another 5 days later, mice were euthanized to harvest lung tissues which were proceeded to viral RNA detection, HE and IHC. **(B)** Viral RNA copies in lung tissues of virus-challenged mice were quantified by one-step SARS-CoV-2 RNA detection kit and plotted as log10 copies per ml (*n* = 4). The RNA copies data represented biological quadruplicate. **(C)** Lung tissues of mice from the saline group and teicoplanin group were proceeded to HE staining and IHC with antibodies against N proteins. **(D)** Schematic of teicoplanin inhibiting the entry of SARS-CoV-2. The SARS-CoV-2 virus binds to the cellular receptor ACE2 by its S proteins which cover the surface of the virus. The S-ACE2 binding event initiates the proteolytic process of TMPRSS2 to S protein on the cellular membrane of CTSL-deficient cells, which triggers the fusion of viral and plasma membranes and subsequently the release of viral genome. Camostat can inhibit the activity of TMPRSS2 and prevent the activation of S proteins, resulting in the inhibition of SARS-CoV-2 infection. In TMPRSS2-deficient cells, SARS-CoV-2 can enter host cells through endocytosis. Within endosomes, CTSL cleaves the S protein and activates the fusion of viral and endosome membranes, resulting in the release of viral genome into the cytoplasm where viruses replicate and assemble. Teicoplanin can effectively block the proteolytic activity of CTSL, rendering the S protein unable to be activated. Without S activation, The SARS-CoV-2 virus is dissolved in the endosome gradually. Scale bars in **(C)** represented 100 μm. Data in **(B)** represented as mean ± SEM in quadruplicate. The *P*-value was calculated by Student’s *t*-test. ***p < 0.001.

To evaluate whether the treatment of teicoplanin could protect individuals from SARS-CoV-2 infection, we conducted mice infection experiments upon teicoplanin treatment. We utilized K18-hACE2 mice which were generated by knocking in the human K18 promoter-driven human ACE2 within the mouse Hipp11 (H11) “safe-harbor” locus. hACE2 mice were intraperitoneally administrated with 100 mg/kg body weight teicoplanin or equal volume of saline, followed by intranasally challenging with 1 × 10^5^ focus-forming units (FFU) of SARS-CoV-2 D614 virus (n = 4 in each group). These mice were euthanized 5 days post infection (Figure 6A). Lung tissues of each mouse were proceeded to SARS-CoV-2 viral RNA quantification, H&E and immunohistochemistry (IHC) analysis. We found that lung tissues of hACE2 mice in saline group harbored large amounts of viral RNA copies (1.9 × 10^4^, 2.3 × 10^5^, 1.2 × 10^5^, and 8.8 × 10^4^ copies per ml for each infected mouse) (Figure 6B). While lung tissues of hACE2 mice in teicoplanin group harbored only few numbers of viral RNA copies (less than 10 copies per ml in average) (Figure 6B). HE and IHC assays also revealed that lung tissues in mice from the saline group were severely damaged upon SARS-CoV-2 challenge, which were interspersed with thickened alveolar septa, collapsed alveoli, and Nucleoprotein (N) protein-expressing cells (Figure 6C). Whereas no pathological changes and N-expressing cells were observed in teicoplanin treatment group (Figure 6C). These results indicated that teicoplanin treatment was able to prevent the infection of SARS-CoV-2 virus in hACE2 mice.

## Discussion

To date, many drugs have been tested for treatment of COVID-19. Remdesivir showed some efficacy in COVID-19 patients, but many severe side effects were observed (Grein et al., 2020). Among patients hospitalized in metropolitan New York with COVID-19, treatment with hydroxychloroquine and/or azithromycin failed to significantly improve in-hospital mortality (Rosenberg et al., 2020). Recently, Merck Sharp and Dohme (MSD) and its partner Ridgeback Biotherapeutics reported that the antiviral drug molnupiravir (MK-4482, EIDD-2801) reduced the risk of hospitalization or death by 50% compared to placebo for patients with mild or moderate COVID-19 based on their Phase III study (NCT04575597). Although previous reports also showed that molnupiravir could prevent SARS-CoV-2 infection and transmission in animal models, more clinical data of long-term monitoring need to be collected to investigate its potential side effects in future clinical trials (Cox et al., 2021; Wahl et al., 2021). Specific treatment for COVID-19 is still lacking and urgently needed. Host cell entry is the first step of the viral life cycle and is an ideal process to develop potential drugs. Several studies and reviews have indicated that antibiotic teicoplanin could be the alternative drug for the treatment of COVID-19 (Baron et al., 2020; Vimberg, 2021). In this study, we conducted many SARS-CoV-2 infection assays and identified that teicoplanin could inhibit the entry of SARS-CoV-2 with an IC_50_ of lower than 2.5 μM. Teicoplanin not only exhibited remarkable inhibitory activity on various pseudotyped SARS-CoV-2 S/HIV-1 mutants’ entry, but also potently restrained the infection of live SARS-CoV-2 viruses including the original D614 reference strain and the later G614 variant. This inhibitory effect was also confirmed by an animal experiment. Therefore, these findings may provide a novel therapeutic treatment to improve current antiviral therapy.

During the invasion phase, SARS-CoV-2 firstly binds to its receptor hACE2 on the surface of host cells. The interaction between the receptor-binding domain (RBD) of the S protein and hACE2 triggers conformational changes within the S protein, which renders the S protein susceptible to be activated by the cellular protease TMPRSS2 (Glowacka et al., 2011; Simmons et al., 2013; Hoffmann et al., 2020). The activation of S proteins by TMPRSS2 results in the fusion of viral membrane and plasma membrane, and subsequently the release of viral genome. While in cells lacking TMPRSS2, the SARS-CoV-2 virus enters to the early endosome of the host cell through endocytosis or macropinocytosis. During the maturation process of the early endosome, the endosome gradually acidifies, which facilitates the entry of viruses into cells. The antiviral drug chloroquine, which can increase the endosomal pH to block virus infection, has been found to inhibit the entry of both SARS-CoV and SARS-CoV-2 (Vincent et al., 2005; Wang et al., 2020; Zhou et al., 2020). During the entry and fusion of SARS-CoV, the cysteine proteinase CTSL within the late endosome can cleave the S protein and activate the membrane fusion, resulting in the release of viral genome (Simmons et al., 2005; Huang et al., 2006).

Several CRIPSR-mediated knock-out and animal infection experiments have highlighted the importance of CTSL in SARS-CoV-2 infection (Wei et al., 2021; Zhao et al., 2021; Zhu et al., 2021). Here we showed that knocking down CTSL potently inhibited SARS-CoV-2 entry. The overexpression of CTSL significantly increased the infectivity of pseudotyped SARS-CoV-2 viruses. Moreover, our data also demonstrated that teicoplanin was able to potently inhibit the infection of both live SARS-CoV-2 viruses and different pseudotyped SARS-CoV-2 mutants by inhibiting the enzymatic activity of CTSL and preventing S proteins activation in TMPRSS2-deficient cells. Without the complete activation of S proteins, the SARS-CoV-2 virus was gradually degraded within the endosome (Figure 6D). Based on all these findings, we believe that the endosomal proteinase CTSL plays vital roles in the infection of SARS-CoV, MERS-CoV, SARS-CoV-2, and possibly other coronaviruses. Therefore, CTSL and its inhibitor teicoplanin provide important therapeutic potential for developing universal anti-CoVs intervention.

Teicoplanin is a glycopeptide antibiotic which is mainly used for serious infection caused by Gram-positive bacteria such as *Staphylococcus aureus* and *Streptococcus* (Parenti et al., 1978; Borghi et al., 1984; Shea and Cunha, 1995). As a commonly used clinical antibiotic, teicoplanin is well known for its low toxicity, mild side effects, long half-life in blood plasma, convenient administration, and high safety. Clinically, the serum concentration of teicoplanin is at least 15 mg/L (8.78 μM) after the completion of the loading dose treatment for most Gram-positive bacterial infections (Zhou et al., 2016). In this study, we found that teicoplanin, and its homolog dalbavancin, could inhibit the entry of SARS-CoV-2 in HEK293T-hACE2^high^ cells expressing the key receptor ACE2. Importantly, teicoplanin was able to inhibit SARS-CoV-2 viruses’ entry with an IC_50_ of 2.038 μM for the Wuhan-Hu-1 reference strain and an IC_50_ of 2.116 μM for the SARS-CoV-2 (D614G) variant, which indicated that teicoplanin inhibited viruses at a relatively low and safe dose. Moreover, our data showed that the pre-treatment of teicoplanin was able to prevent SARS-CoV-2 infection in hACE2 mice. Given that the principles of antiviral therapy are to prevent virus infection and use extensively as early as possible, it is reasonable to recommend the use of teicoplanin for SARS-CoV-2 in the early infection stage. Therefore, teicoplanin could potentially function as a dual inhibitor for both SRS-CoV-2 and co-infected Gram-positive bacteria.

## Data Availability Statement

The original contributions presented in the study are included in the article/Supplementary Material, further inquiries can be directed to the corresponding author/s.

## Ethics Statement

The animal study was reviewed and approved by the Ethics Committees of Guangdong Provincial People’s Hospital and Sun Yat-sen University.

## Author Contributions

XM initiated the study, supervised the project, and wrote the manuscript. FY, TP, FH, and XM designed the experiments and analyzed the data. FY, TP, FH, and XM performed the experiments. RY, JL, HF, JZ, WL, YL, YY, TY, RL, XZ, XL, QC, AL, FZ, BL, FyH, XT, LL, KD, XH, HZ, and YZ helped with some experiments. FY and XM proofread the whole manuscript. FY, TP, FH, and XM wrote, reviewed, and edited the manuscript. All authors contributed to the article and approved the submitted version.

## Conflict of Interest

The authors declare that the research was conducted in the absence of any commercial or financial relationships that could be construed as a potential conflict of interest.

## Publisher’s Note

All claims expressed in this article are solely those of the authors and do not necessarily represent those of their affiliated organizations, or those of the publisher, the editors and the reviewers. Any product that may be evaluated in this article, or claim that may be made by its manufacturer, is not guaranteed or endorsed by the publisher.

## Footnotes

1
https://www.ncbi.nlm.nih.gov/labs/virus/vssi/#/

2
https://www.gisaid.org/

3
https://bioedit.software.informer.com/7.2/
