# Supplementary material for: Glycopeptide Antibiotic Teicoplanin Inhibits Cell Entry of SARS-CoV-2 by Suppressing the Proteolytic Activity of Cathepsin L
Source: Front Microbiol. 2022 Apr 28;13:884034. doi: 10.3389/fmicb.2022.884034 (PMC9096618; doi:10.3389/fmicb.2022.884034)
Supplement: Supplementary file 1 [file Data_Sheet_1.docx]

## Supplementary Figures


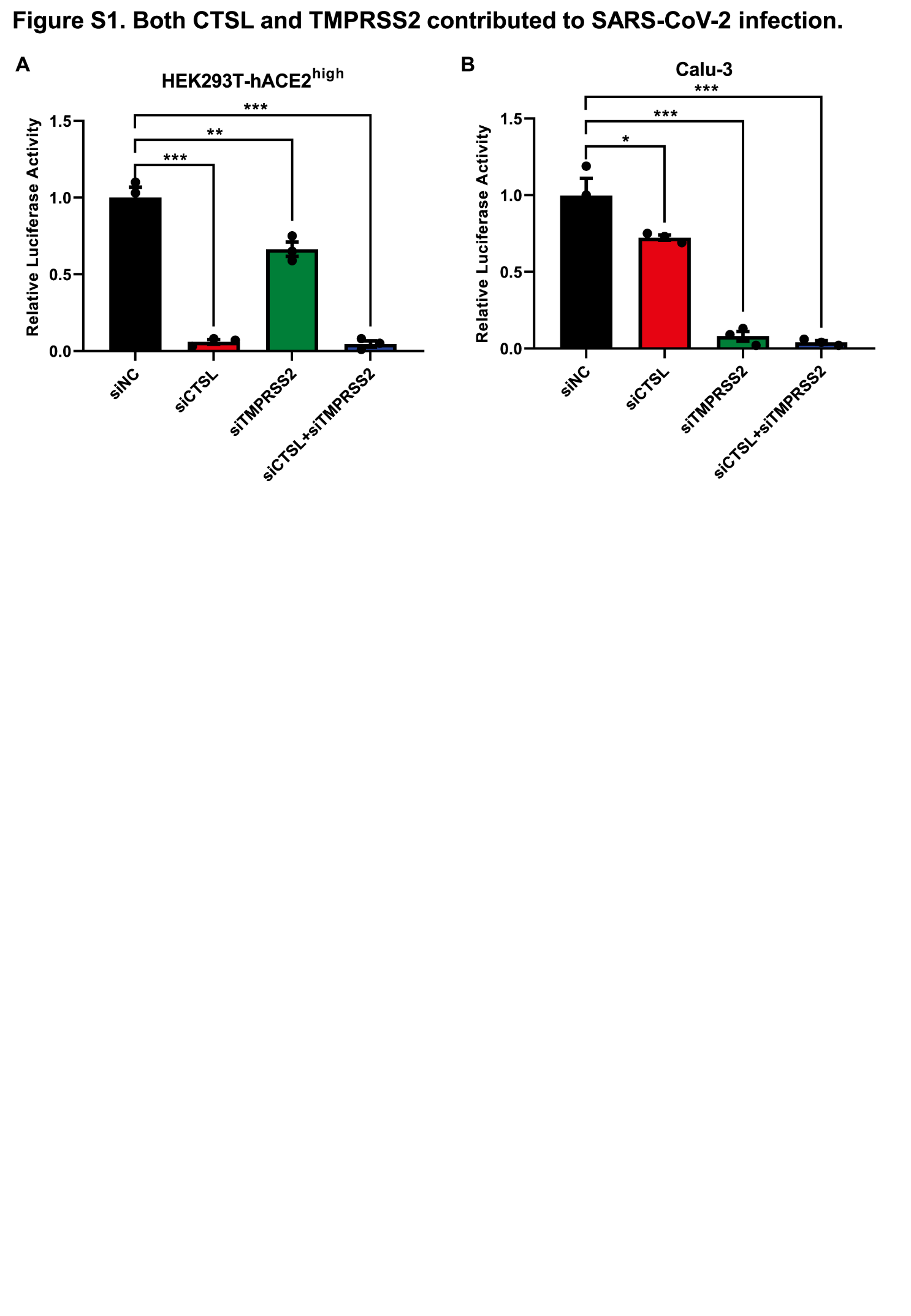


**Supplemental Figure 1. Both CTSL and TMPRSS2 contributed to SARS-CoV-2 infection.** (**A**) HEK293T-hACE2^high^ cells were transfected with siRNAs targeting CTSL or TMPRSS2, followed by infecting with pseudotyped SARS-CoV-2 S / HIV-1 virus. Another 48 hours post infection, the amounts of luciferase within cells were measured and represented as relative luciferase activity (n=3). (**B**) Calu-3 cells were transfected with siRNAs targeting CTSL or TMPRSS2, followed by infecting with pseudotyped SARS-CoV-2 S / HIV-1 virus. The relative expression of luciferase was calculated as in (**A**) (n=3). Data represented as mean ± SEM in triplicate. P-values were calculated by one-way ANOVA with Dunnett’s multiple comparison test which compared the mean of each group with the mean of the control group. *p < 0.05, **p < 0.01, ***p < 0.001.

**
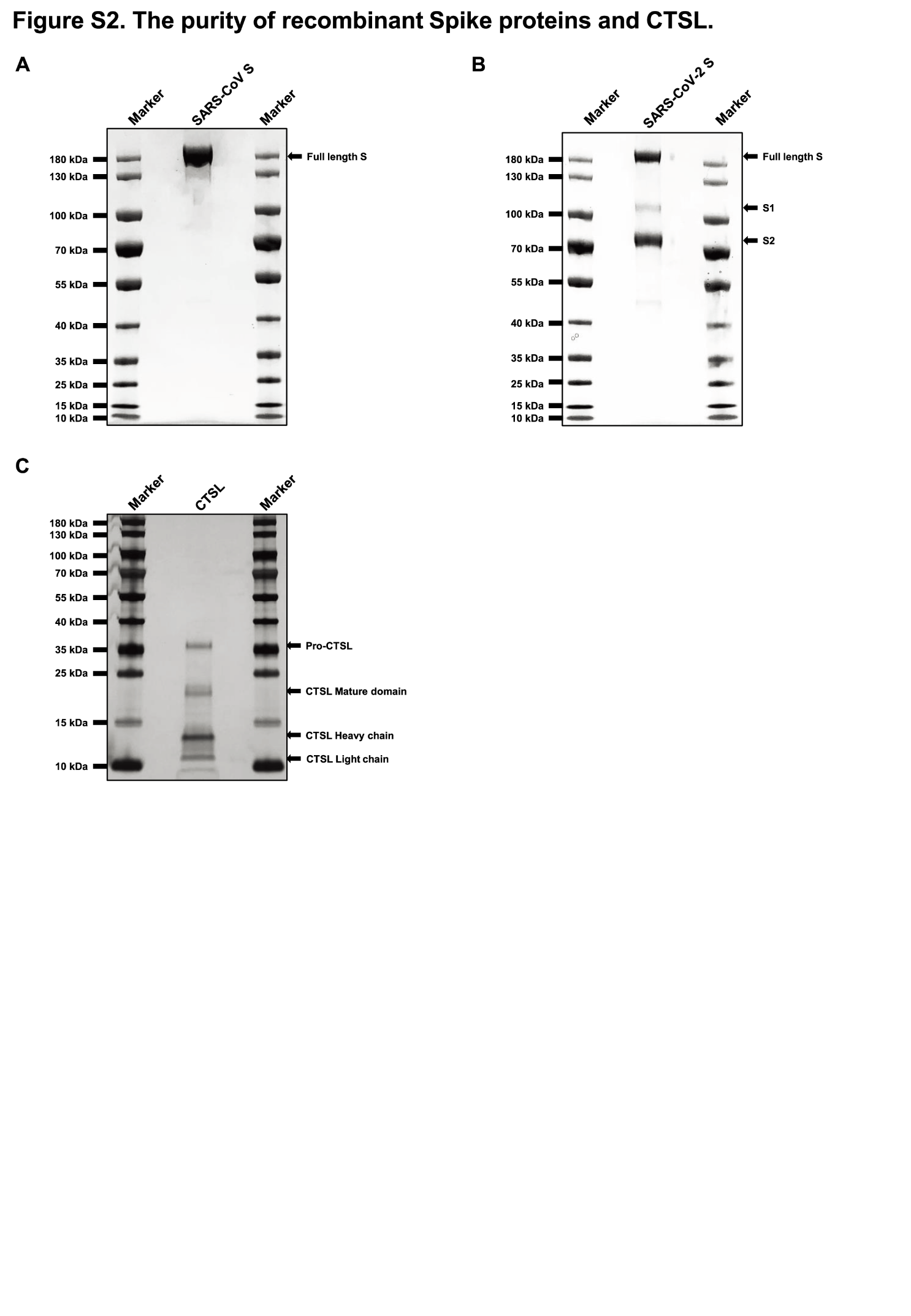
**

**Supplemental Figure 2. The purity of recombinant Spike proteins and CTSL.** (**A-C**) The purities of recombinant SARS-CoV Spike (**A**), SARS-CoV-2 Spike (**B**) and CTSL (**C**) were analyzed by silver staining. Of note, the purified SARS-CoV-2 Spike proteins contained both full-length and Furin-precleaved Spike, resulting in the appearance of full-length S, S1 and S2 proteins. The purified CTSL proteins contained pro-CTSL, CTSL mature domain, CTSL heavy chain and CTSL light chain.
